# Metabolite Profiling of the Indian Food Spice Lichen, *Pseudevernia furfuracea* Combined With Optimised Extraction Methodology to Obtain Bioactive Phenolic Compounds

**DOI:** 10.3389/fphar.2021.629695

**Published:** 2021-05-10

**Authors:** Rishu Kalra, Xavier A. Conlan, Carlos Areche, Rahul Dilawari, Mayurika Goel

**Affiliations:** ^1^ TERI-Deakin Nanobiotechnology Centre, Sustainable Agriculture Division, The Energy and Resources Institute, Gurugram, India; ^2^ Centre for Chemistry and Biotechnology, School of Life and Environmental Sciences, Deakin University, Geelong, VIC, Australia; ^3^ Departamento de Química, Facultad de Ciencias, Universidad de Chile, Nuñoa, Chile; ^4^ CSIR-Institute of Microbial Technology, Chandigarh, India

**Keywords:** lichen, antioxidants, atraric acid, olivetoric acid, UHPLC-MS, metabolomics profiling, *Pseudevernia furfuracea*, spatial localization

## Abstract

*Pseudevernia furfuracea* (L.) Zopf *(Parmeliaceae)* is a well-known epiphytic lichen commonly used in Indian spice mixtures and food preparations such as curries. This study is an attempt to find the best extraction methodology with respect to extractive yield, total polyphenolic content (TPC), total flavonoid content and antioxidant activities of lichen *P. furfuracea*. Two phenolic compounds, atraric acid and olivetoric acid were isolated and quantified in their respective extracts with the aid of reverse phase high performance liquid chromatography (RP-HPLC). The highest concentration of both the compounds, atraric acid (4.89 mg/g DW) and olivetoric acid (11.46 mg/g DW) were found in 70% methanol extract. A direct correlation was also observed between the concentrations of these compounds with the free radical scavenging potential of the extracts which might contribute towards the antioxidant potential of the extract. Moreover, scanning electron microscopy and HPLC analysis which was used to study the effect of pre-processing on extraction process highlighted the capacity of a mixer grinder technique for improved separation of surface localized metabolites and enrichment of the fraction. An investigation of the chemical profile of the bioactive extract 70% methanol extract using UHPLC-DAD-MS lead to tentative identification of forty nine compounds. This extract was also assessed towards HEK 293 T cell line for cytotoxicity analysis. Concentration range of 0.156 to 100 µg/ml of PF70M extract exhibited no significant cell death as compared to control. Further, the active extract showed protective effect against hydroxyl radical’s destructive effects on DNA when assessed using DNA nicking assay. Based upon this, it can be concluded that optimization of extraction solvent, sample pre-proceesing and extraction techniques can be useful in extraction of specific antioxidant metabolites.

## Introduction

Bioactive secondary metabolites of natural origin are extremely useful in food, pharmaceutical, agrochemical, nutraceutical and cosmeceutical industries due to their multifaceted biological activities such as antioxidant, antimicrobial, anticancer and antifungal (Goel et al., 2011; Sisodia et al., 2013; Goel et al., 2014; Newman and Cragg, 2016; Goel et al., 2020). Related to this, natural product derived antioxidants have been shown to exhibit strong protective effects against a variety of chronic health problems by postponing the damage caused by oxidative stress (Liu 2003). Antioxidants and their associated benefits is currently a subject of intensive research due to an increase in lifestyle disorders associated with stress and in line with this some lichens have been explored for their antioxidant potential through the last decade (Manojlovic et al., 2012; Kosanic et al., 2013; Kosanić et al., 2014; Kumar et al., 2014; Zugic, 2016).

Lichens are the microbial association defined by a stable symbiotic relationship between a mycobiont (fungal partner) and a photobiont (photoautotrophic partner, usually a green alga or cyanobacterium) (Calcott et al., 2018). Traditionally, some of the lichens are consumed for their culinary qualities, and used for their preservative and medicinal properties (Upreti et al., 2005). *Pseudevernia furfuracea* is well-known foliose lichen, commercially used in the spice mixture, food preparation like curries and as preservative in food and herbal preparations (Güvenç et al., 2012; Kosanic et al., 2013; Aoussar et al., 2017). Despite a decade of study, the potential of lichens to yield novel unique metabolites have not been fully realised. However an increase in technological capacity has recently rekindled the attention of pharmaceutical industries and researchers to determine potential of lichens antioxidants (Crittenden, 1991; Nash, 1996). The ethnopharmacological importance and consumption of the lichens as a functional food has resulted in investigations focusing upon the discovery and identification of the naturally occurring compounds responsible for their bioactivity. Phenolic polyketides present in lichens have been reported to have strong antioxidant properties (Hidalgo et al., 1994; Odabasoglu et al., 2006). Moreover, it has been well documented that extraction procedures used to isolate these compounds are vital for determining extractive yield and total polyphenols and thus antioxidant potential of the extracts (Spigno and De Faveri, 2007; Ismail et al., 2019). Factors such as extraction solvents, sample processing techniques, particle size, extraction techniques have been shown to have an influence on extraction efficiency and subsequent bioactivity (Cacace and Mazza, 2003; Ng et al., 2012). Therefore, it was rationalized that the extraction procedure is an important prerequisite for the comprehensive exploration of the beneficial effects of the species.

This study is designed to determine the effect of different extraction solvents, different extraction techniques and different grinding methods on obtaining polyphenolic and flavonoid rich fractions and evaluation of their antioxidant potential from lichen. The purpose of the study was to find best extraction methodology with respect to extractive yield and antioxidant activities of obtained extracts in order to inform the food industry at large. The main factors selected for their presumed influence on the extraction efficiency and antioxidant potential are polarity of solvent, grinding or processing method and extraction methods. Separation of the metabolites rich portion from the lichen matrix was afforded by a grinding method prior to the determination of spatial localization of metabolites responsible for bioactivity in sample. Moreover, two key metabolites were isolated and quantified in different extracts and was found to be directly correlated with the free radical scavenging potential. In order to fully understand the fundamental process which influences the sample processing techniques, scanning electron microscopy was used to identify the separated fractions obtained after grinding and residual components left after extraction process. Finally, UHPLC-DAD-MS was used to study the qualitative composition of active extract and as such this work forms a platform for the advancement of lichen based food in future products and to enable a better understanding of food research where lichen forms a part of the diet.

## Materials and Methods

### Material

**FIGURE 1:**
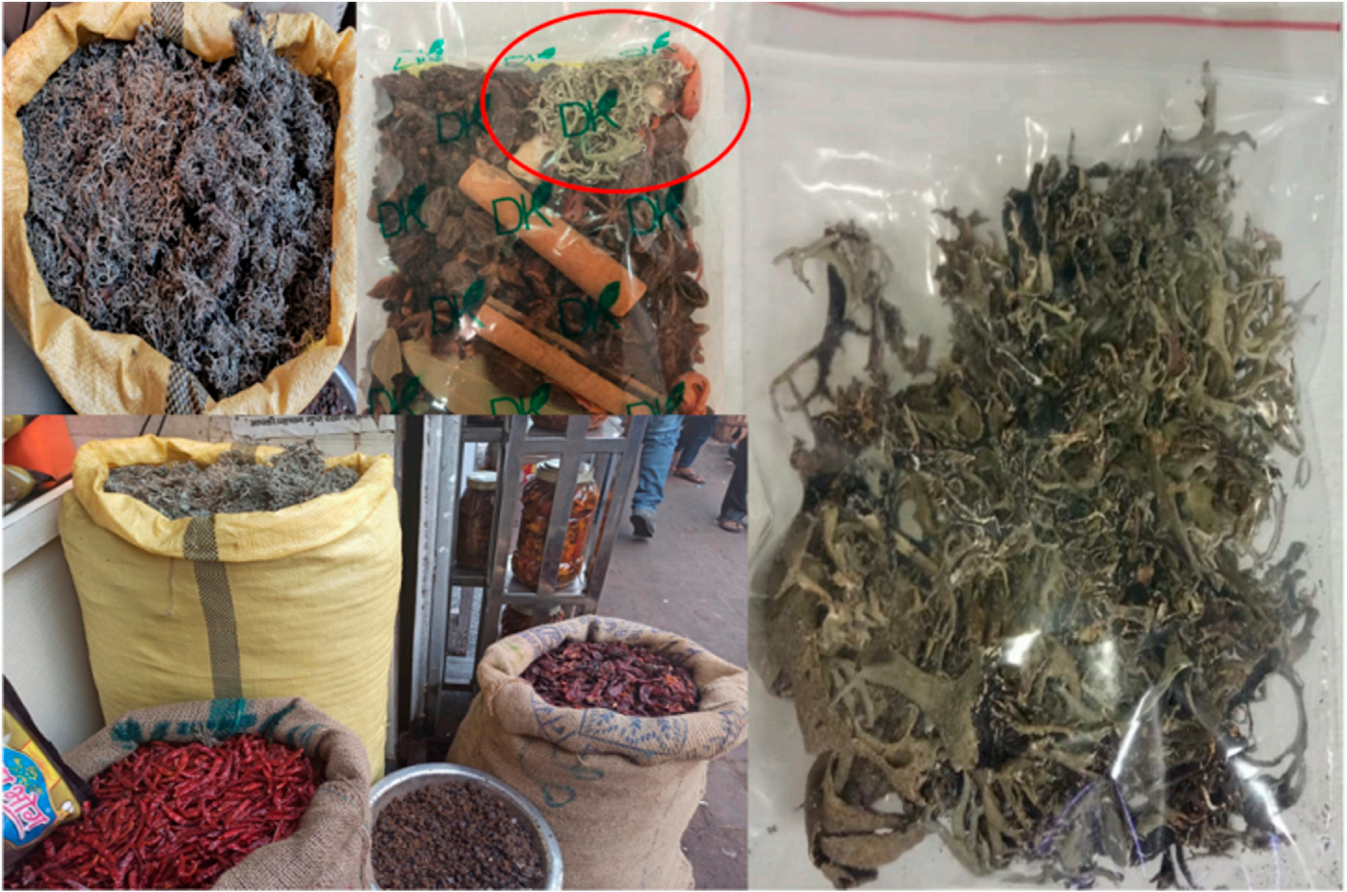
*Pseudevernia furfuracea* as it is sold in the spice market khari baoli (New Delhi, India).

Lichen material was collected from a local vendor (Khari baoli, New Delhi, India) in November 2017. Khari baoli market is one of Asia’s largest wholesale spice market selling all kinds of spice. Sample *Pseudevernia furfuracea* is being sold as a part of spice mixture (Figure 1). The sample was then washed in distilled water, dried at room temperature (25 ± 5°C). The sample was identified by Prof. Prem Lal Uniyal and a voucher specimen (DUH 1401) was deposited in the herbarium of University of Delhi, Delhi, India.

### Chemicals and Reagents

2,2-Diphenyl-1-picryhydrazyl (DPPH) radical, 2,2-azinobis (3-ethylbenzothiazoline-6-sulphonic acid) (ABTS), folin-Ciocalteu reagent, gallic acid, quercetin, aluminum chloride anhydrous, 2-thiobarbituric acid (TBA), deoxyribose, ethylenediaminetetraacetic acid (EDTA) disodium dihydrate, ascorbic acid, alamar blue and trolox were procured from Sigma–Aldrich (Sigma Aldrich India Pvt Ltd., Bangalore, India). Potassium persulphate and sodium bicarbonate were purchased from Sisco Research Laboratories Pvt. Ltd. (Maharashtra, India). Ferric chloride, hydrogen peroxide, orthophosphoric acid were purchased from Fischer Scientific (Mumbai India). All the solvents methanol, acetone, hexane, ethyl acetate, dichloromethane, and dimethyl sulphoxide were purchased from Merck Millipore (KGaA, Darmstadt, Germany). Chemicals and solvents used were of analytical and HPLC grade, respectively.

### Grinding of the Lichen Samples

The raw sample was dried in an oven at 40°C before grinding and divided into two portions; the first portion of sample (200 g) was chopped with the help of scissors to get fine pieces. These fine pieces were then crushed with the help of pestle mortar and resulted into homogenised powder. Other portion of the raw sample (50 g) was ground up with a mixer-grinder (Philips, HL-1606) at maximum rotation speed for 15 min. This process led to the separation of sample into two portions, cortex powder and medulla pieces. Both the fractions were analysed and tested separately for their surface morphology, bioactive contents and bioactivity assays.

### Extraction of Lichen Samples

**FIGURE 2:**
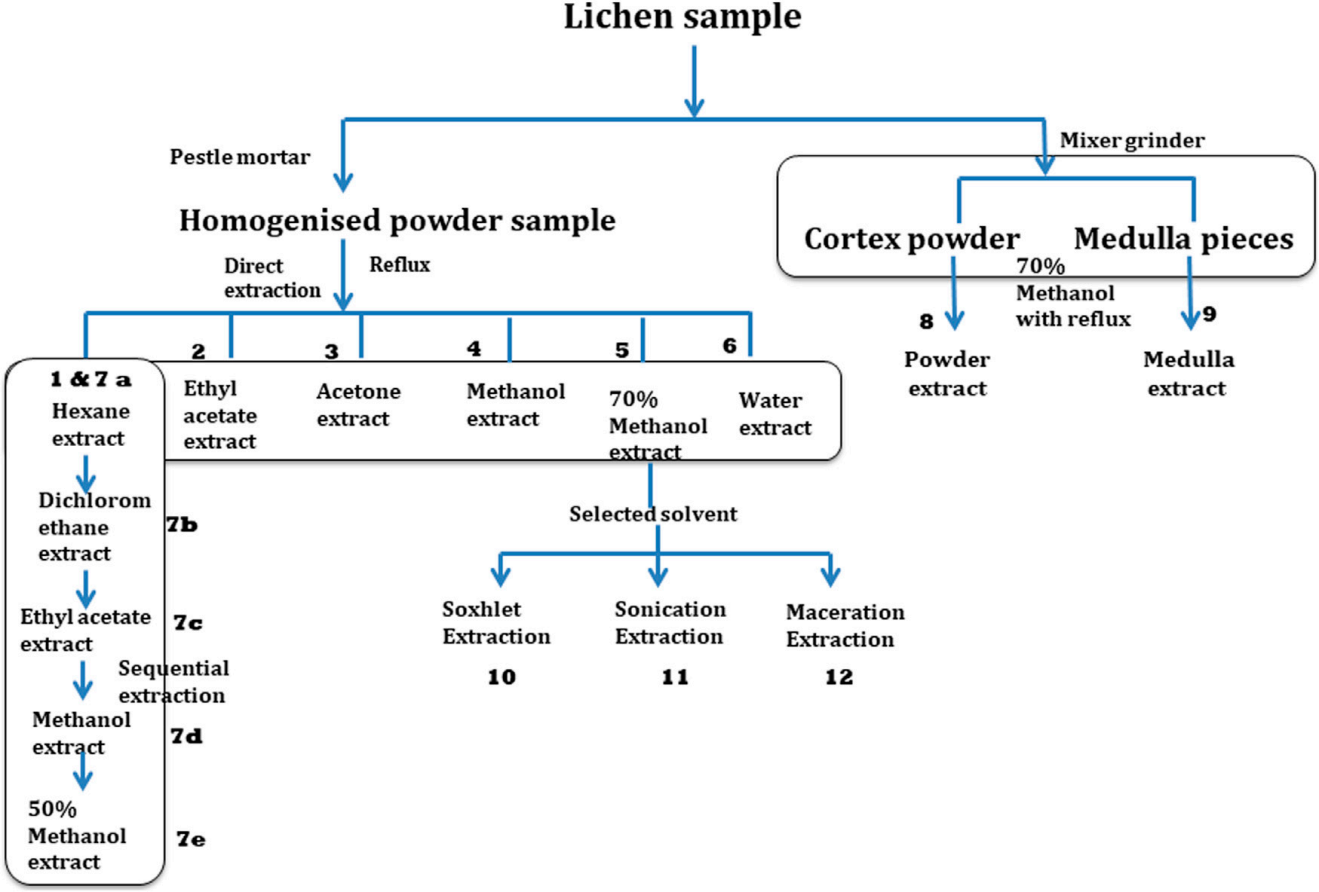
Experimental design for the preparation of different extracts.

Lichen sample was extracted using three variables parameters which include different extraction solvents, different grinding methods and different extraction methods. Figure 2 explaining the complete experimental design for the preparation of different extracts gives a clear overview of all the different extraction.

To analyse the effect of different extraction solvent, direct extraction was done using hexane, ethyl acetate, acetone, methanol, 70% methanol, water. On the other hand, in case of sequential extraction, solvents of increasing polarity (hexane followed by dichloromethane, ethyl acetate, methanol and 50% methanol) were used in serial manner (Figure 2). To understand the effect of pre-processing on extraction process, differently grounded sample as mentioned above (*Grinding of the Lichen Samples*) were extracted separately using optimized solvent (70% methanol) and reflux method. All the extractions were done in triplicate using reflux apparatus at 60°C. Similarly, different extraction methods including maceration, sonication, reflux and soxhlet were used to access the effect of methodology on extraction yield and metabolic profiling.

Extraction yield in all the methods was calculated by following formula:

$$\text{Total}\;\text{Yield}\;\left(\%\right)=\left(\frac{\text{Total}\;\text{extract}\;\text{mass}}{\text{Mass}\;\text{of}\;\text{lichen}}\right)\;\times \;100$$

### Determination of Total Polyphenol and Total Flavonoid Contents

Total polyphenol content (TPC) and total flavonoid content (TFC) of the lichen extracts were measured using previously reported Folin-Ciocalteu’s method (Singleton Vernon et al., 1999) and aluminum chloride method (Ordonez et al., 2006), respectively. The total polyphenol content of the extracts was expressed as mg of gallic acid equivalent (GAE)/g of dry lichen material on the basis of calibration curve of gallic acid (20–200 µg/ml; *R* ^2^ = 0.99). The total flavonoid content of the extracts was expressed as mg of quercetin equivalent (QE)/g of dry lichen material on the basis of calibration curve of quercetin (1–100 µg/ml; *R* ^2^ = 0.99). The absorbance for each methodology was determined using UV-Visible spectrophotometer-2450 (Shimadzu).

### Evaluation of Free Radical Scavenging Potential

Free radical scavenging potential of the extract were evaluated by ABTS^**+**^ radical cation decolourisation assay, DPPH radical scavenging assay, hydroxyl radical scavenging potential using trolox as the standard.

#### ABTS Radical Scavenging Potential

The ABTS assay was performed in line with the previously described protocol by Re *et al.*, with slight modification as follows (Re et al., 1999). The ABTS^**+**^ radical cation was prepared by mixing 7 mM ABTS aqueous solution with 2.45 mM potassium persulfate (final concentration) and allowing the mixture to stand in the dark at room temperature for 12–16 h before use. The working solution was prepared by diluting the stock solution with ethanol for an initial absorbance of about 0.70 ± 0.02 at 745 nm. Free radical scavenging potential was assessed by mixing different concentration (5–100 µg/ml) of sample and standard Trolox (1–20 μM; 0.25–5 µg/ml) with ABTS^**+**^ working standard to make a final volume of 1 ml. The decrease in absorbance was measured exactly after 6 min at 30°C. The half maximal inhibitory concentration (IC_50_) for test samples and Trolox was calculated by plotting the scavenging capacity against the concentration. The results were expressed as Trolox Equivalent Antioxidant Capacity (TEAC) µM Trolox/g DW.

#### 1, 1-Diphenyl-2-picrylhydrazyl Radical-Scavenging Potential

Free radical scavenging potential of the samples was tested using a previously developed DPPH radical-scavenging protocol (Brand-Williams et al., 1995). The 0.2 mM solution of DPPH was prepared in 70% methanol and stirred overnight in the dark at room temperature before use. Free radical scavenging potential was assessed by mixing different concentration (10–300 µg/ml) of samples and standards Trolox (5–50 μM; 1.25–12.5 μg/ml) with DPPH solution to make a final volume of 1 ml. These samples were shaken well and kept in dark for 30 min at room temperature. The decrease in absorbance was measured (at room temperature) after 30 min at 517 nm. The results were expressed as Trolox Equivalent Antioxidant Capacity (TEAC) µM Trolox/g DW as mentioned above in ABTS assay.

#### Hydroxyl Radical Scavenging Potential

This assay was performed by previously described protocol described by Li (2013). Hydroxyl radical was generated by the Fenton reaction (Fe^3+^-ascorbate-EDTA-H_2_O_2_ system). Samples (10–50 μg/ml) and standard Trolox (1–25 μM; 0.25–6.25 μg/ml) were mixed with 400 µL of phosphate buffer (0.2 M, pH 7.4) followed by 50 µl of deoxyribose (50 mM), 50 µl of Na_2_EDTA (1 mM), 50 µl of FeCl_3_ (3.2 mM) and 50 µl of H_2_O_2_ (50 mM). Addition of 50 µl of ascorbic acid (1.8 mM) initiates the reaction. Total volume of the reaction mixture was adjusted to 800 µl with buffer. The reaction mixture was incubated at 50°C for 20 min followed by addition of 250 µl of trichloroacetic acid (10%, w/w) for the termination of reaction. Chromogen was developed by addition of 150 µl of TBA (5%, in 1.25% NaOH aqueous solution) and incubated at 105°C for 15 min. The mixture was cooled and absorbance was measured at 532 nm. The results were expressed as Trolox Equivalent Antioxidant Capacity (TEAC) µM Trolox/g DW as mentioned above in ABTS assay.

### Antioxidant Activity Using DNA Nicking Assay

Antioxidant activity of bioactive extract was also assessed by DNA damage protection assay. Analysis was performed using supercoiled pBSK plasmid DNA according to the method of Zhao et al. (2014) with slight modifications (Zhao et al., 2014). A mixture of plasmid DNA (0.5 μg) and active extract PF70M in concentration range of 25–200 μg/ml was incubated at room temperature for 10 min followed by addition of equal volume of Fenton's reagent (30 mM H_2_O_2_, 80 mM FeCl_3,_ and 50 mM ascorbic acid). Reaction mixtures were then allowed incubated for 30 min at 37°C. The DNA was examined on 1% agarose gel using ethidium bromide staining. Curcumin was found as positive control.

### Isolation and Quantification of Metabolites by High Performance Liquid Chromatography

The isolation of metabolites was performed using column chromatography (CC) over silica gel (100–200#). The extracts was eluted using hexane/ethyl acetate (95:5 to 70:30, v/v) as eluent and yielded nine major fractions (A-I). Fraction B was further subjected to CC over silica gel (60–120#), using hexane/ethyl acetate (95:5 to 28:20, v/v) lead to isolation of pale yellow crystals (compound 1). Fractions F was subjected to preparative TLC and lead to isolation of pale yellow amorphous powder (compound 2). Both the isolated compounds were identified with the help of NMR and mass spectral analysis.

Chemical analysis of the lichen extracts was performed using a Waters HPLC system consisting of a 600 quaternary gradient pump with an online vacuum degasser, a 717 auto-sampler, and 2996 diode array detector. Separation of the compounds of interest was achieved using a reversed phase C_18_Luna column (Phenomenex, Lorance, CA, United States, 150 mm × 4.6 mm, 5 μm) and a 10 µl injection volume. A five point calibration curve from standard solutions was prepared by 10 µl injections of 5–100 μg/ml. Commercial standards of lichen compounds were not available therefore abovementioned isolated compounds were used as standards for the quantification. An HPLC gradient was applied: A (0.8% orthophosphoric acid in water) and B (acetonitrile). The following gradient was used at a flow rate of 1 ml/min: initial, 100% A in 0–5 min; 100% B in 5–45 min, 100% A in 45–60 min. Presence of compounds in the extracts was confirmed by comparison of retention time and ultraviolet and visible (UV-Vis) absorption spectra (Chowdhary et al., 2019).

### UHPLC-DAD-MS Analysis

A Thermo Scientific Dionex Ultimate 3000 UHPLC system hyphenated with a Thermo high resolution Q Exactive focus mass spectrometer (Thermo, Bremen, Germany) were used for analysis. Mass calibration for the Orbitrap™ was performed once a week, in both negative and positive modes, to warrant a working mass accuracy lowers than or equal to 5 ppm. UHPLC and mass parameters were used as per previously described method for the analysis of lichen samples (Salgado et al., 2018). An HPLC gradient having eluent (A) 0.1% formic acid in water, eluent (B) 0.1% formic acid in acetonitrile was performed. The following gradient was used at a flow rate of 1 ml/min: 5% B in 0–5 min; 30% B in 5–10 min, 30% B in 15 min, 70% B in 20 min, 70% B in 25 min, 5% B in 35 min, 12 min for column equilibration before each injection. The injection volume was 10 µL. Analysis was performed on UHPLC C_18_ column (Acclaim, 150 mm × 4.6 mm ID, 2.5 mm, Thermo Fisher Scientific, Bremen, Germany) operated at 25°C. The MS conditions were as follows: Spray voltage 2500 V (for ESI-); aux. gas unit flow rate 20; aux gas heater temperature 500°C; capillary temperature 400°C; sheath gas flow rate 75 units. Full scan range was set in negative mode with the resolving power of 70,000 FWHM (full width half maximum) at *m/z* 200. For the compounds of interest, a scan range of *m/z* 100–1000 was chosen; the automatic gain control (AGC) set at 3 × 10^6^ and the injection time set to 200 ms. Scan-rate was set at 2 scans s⁻^1^. A mixture of taurocholic acid sodium salt, buspyrone hydrochloride, and sodium dodecil sulfate (Sigma-Aldrich, Darmstadt, Germany), plus Ultramark 1621 (Alpha Aezar, Stevensville, MI, United States) dissolved in a mixture of acetic acid, acetonitrile, water and methanol, was used as calibration solution to ensure a working mass accuracy lower than or equal to 5 ppm. For confirmation purposes, a targeted MS/MS analysis was performed using the mass inclusion list, with a 30 s time window, with the Orbitrap spectrometer operating in negative mode at 17,500 FWHM (*m/z* 200). The AGC target was set to 2 × 10^5^, with the maximum injection time of 20 ms. The precursor ions were filtered by the quadrupole which operates at an isolation window of *m/z* 2. The fore vacuum, high vacuum and ultrahigh vacuum were maintained at approximately 2 mbar, from 105 and below 1010 mbar, respectively. Collision energy (HCD cell) was operated at 30 kv. Detection was based on calculated exact mass of target compounds, as shown in Table 3.

### Cell Cytotoxicity/Viability Assay

The cytotoxicity for most bioactive extract (PF70M) extract was assessed toward HEK 293 T cell line using Alamar Blue (Resazurin), a cell metabolic activity reagent (Sigma) (Rampersad et al., 2012). The log phase cells were harvested and cell count was adjusted to (1 × 10^4^/well) in DMEM containing 10% fetal bovine serum (FBS) and incubated for 12 h under 5% CO_2_ at 37°C in 96-well microplates. Next day, cells were incubated with varying concentrations of PF70M extract at 37°C for 24 h. Following this, 0.02% Alamar blue reagent was added and the cells were further incubated for 6–8 h under 5% CO_2_ at 37°C. Fluorescence was measured with excitation wavelength at 545 nm and emission wavelength at 590 nm in Elisa plate reader (Power Wave HT Microplate Spectrophotometer–BioTek).

The percent difference between treated and un-treated cells was calculated by following formula:

$$\mathrm{\%Viability}=\left(\frac{\text{RFU}\;\text{of}\;\text{treated}\;\text{sample}}{\text{RFU}\;\text{of}\;\text{untreated}}\right)\times 100$$

Where RFU stands for relative fluorescence unit.

### Statistical Analysis

Results are expressed as mean of triplicate data ± standard error and Pearson correlation between polyphenol contents, flavonoid content, atraric acid and olivetoric acid concentration and antioxidant potential was established by IBM SPSS statistical software.

## Results and Discussion

### Effect on Extractive Yield

#### Different Solvent Treatment

The percentage extractive yield obtained from the solvent treatments ranged from 1.38 ± 0.44 to 15.19 ± 0.24% and the full data can be viewed in Table 1. The maximum extractive yield was obtained in 70% methanol followed by water and methanol and the key driver behind the increased extraction efficiency with 70% methanol is principally due to extraction of wide range of compounds (polar to non-polar) present in *Pseudevernia furfuracea*. The optimum extraction yield observed from the successive extraction is likely due to the better mass transfer from the substrate, which is clearly visible in the scanning electron microscopy (SEM) image (Supplementary Figure S1). The residue (a dense layer of platelets like crystals) left after each extraction can be observed and these crystals were washed off successively after each extraction. These findings suggest better extraction efficiency from the substrate and thus the driver of the highest extractive yield in the successive extraction (Supplementary Figure S1). Nevertheless, taking into consideration, other factors such as time, resources required and nature of metabolites directed toward bioactivity, 70% methanol was found to be the most appropriate solvent for the efficient and rapid extraction of bioactive phenolic constituents.

**TABLE 1:** Extraction yield, total phenolics and antioxidant potential of *P. furfuracea* using different extraction solvents, grinding method and extraction method.

Experiment number	Extraction solvent/method/grinding technique	Extractive yield ±SE (wt%)	TPC^a^ (mg GAE/g DW)	TFC^b^ (mg QE/g DW)	TEAC^c^ (µM TROLOX/g DW)	Atraric acid^d^ (mg/g DW)	Olivetoric acid^d^ (mg/g DW)
1 and 7a	Hexane/reflux/pestle mortar	1.38 ± 0.44	0.70 ± 0.10	0.02 ± 0.00	1.66 ± 0.00* 0.45 ± 0.01** 19.33 ± 0.21***	Nd	Nd
2	Acetone/reflux/pestle mortar	4.04 ± 0.32	5.92 ± 0.38	0.22 ± 0.04	6.91 ± 0.16* 5.33 ± 0.07** 99.12 ± 1.14***	0.63	1.95
3	Ethylacetate/reflux/pestle mortar	3.7 ± 0.44	9.80 ± 0.60	0.10 ± 0.00	7.62 ± 0.04* 4.65 ± 0.06** 64.77 ± 0.74***	1.20	1.44
4	Methanol/reflux/pestle mortar	7.83 ± 0.68	21.43 ± 0.11	0.24 ± 0.00	25.97 ± 0.69* 13.94 ± 0.26** 260.31 ± 7.60***	0.72	2.56
5	70% methanol/reflux/pestle mortar	9.81 ± 0.41	32.38 ± 0.29	0.38 ± 0.00	38.30 ± 1.53* 19.10 ± 0.60** 353.06 ± 9.58***	2.41	11.46
6	Water/reflux/pestle mortar	9.55 ± 0.95	5.84 ± 0.10	0.16 ± 0.01	5.22 ± 0.00* 3.02 ± 0.09** 80.30 ± 5.47***	0.08	Nd
7	**Sequential extraction**	**15.19** ± **0.24**					
7b	Dichloro methane/reflux/pestle mortar	2.64 ± 0.27	5.39 ± 0.10	0.07 ± 0.00	7.10 ± 0.04* 2.45 ± 0.07** 42.03 ± 0.19***	0.15	0.42
7c	Ethyl acetate/reflux/pestle mortar	2.23 ± 0.26	5.88 ± 0.07	0.03 ± 0.00	4.96 ± 0.15* 2.96 ± 0.03** 32.56 ± 0.79***	0.14	0.69
7d	Methanol/reflux/pestle mortar	4.9 ± 0.11	10.65 ± 0.11	0.06 ± 0.00	13.39 ± 0.24* 4.81 ± 0.11** 75.08 ± 1.42***	0.97	3.39
7e	50% methanol/reflux/pestle mortar	4.28 ± 0.56	3.86 ± 0.08	0.11 ± 0.00	6.12 ± 0.02* 2.40 ± 0.05** 59.16 ± 1.00***	0.43	0.69
8	70% methanol/reflux/mixer grider (cortex powder portion)	8.92 ± 0.08	28.07 ± 0.16	0.35 ± 0.00	23.90 ± 0.05* 14.06 ± 0.13** 302.39 ± 6.40***	2.61	6.69
9	70% methanol/reflux/mixer grider (medulla pieces)	5.25 ± 0.19	14.87 ± 0.03	0.11 ± 0.00	15.26 ± 0.09* 6.52 ± 0.03** 99.32 ± 1.76***	1.01	6.49
10	70% methanol/soxhlet/pestle mortar	11.06 ± 0.26	41.73 ± 2.37	0.44 ± 0.00	47.57 ± 0.16* 22.48 ± 0.04* 388.33 ± 3.31***	4.89	8.35
11	70% methanol/sonication/pestle mortar	6.72 ± 0.42	18.39 ± 0.48	0.14 ± 0.00	20.16 ± 0.03* 7.38 ± 0.08** 127.58 ± 1.22***	4.04	6.03
12	70% methanol/maceration/pestle mortar	7.04 ± 0.30	17.54 ± 0.00	0.12 ± 0.00	23.28 ± 0.08* 8.25 ± 0.14** 176.15 ± 1.13***	0.21	6.83

^a^ Data expressed as mg of gallic acid equivalent (GAE)/g of lichen dry material.

^b^ Data expressed as mg of quercetin equivalent (QE)/g of lichen dry material.

^c^ Data expressed as µM of Trolox equivalent/g of lichen dry material (*) TEAC assayed by ABTS method, (**) TEAC assayed by DPPH method, (***) TEAC assayed by OH scavenging method.

^d^ Amount represented in µg/g of the dry lichen material, nd-peak not detected.

#### Different Grinding Techniques

As lichen metabolites are not homogeneously distributed in the sample matrix, different types of grinding methods or sample processing technique may influence extraction yield, TPC, metabolites concentration and subsequently the antioxidant potential of extract. To understand this effect, two different grinding methods, a pestle mortar and a mixer grinder were assessed. Samples crushed in mortar pestle resulted in a homogenised powder and was considered as a whole thallus sample for extraction, represented in experiment 5 (Figure 2) affording the extraction yield of 9.81 ± 0.41%. Whereas the total extractive yield obtained from the mixer grinder method (14.17 ± 0.19%) which is the sum total of extractive yield of cortex powder sample (8.92 ± 0.08%) and medulla pieces (5.25 ± 0.11%) is far more than the yield obtained from sample ground using mortar pestle which generated the homogenous powder.

#### Different Extraction Techniques

The extraction techniques chosen for their putative influence on extraction efficiency with a single optimized solvent were identified as soxhlet, reflux, sonication and maceration. With the optimum solvent (70% methanol), the extractive yield was found to be best with soxhlet extraction (11.03 ± 0.26%), followed by reflux (9.81 ± 0.41%), then maceration (7.04 ± 0.30%) and finally sonication (6.72 ± 0.42%). The higher yield obtained with the soxhlet extraction may be attributed to the application of warm solvent and exhaustive extraction with fresh solvent in every siphon.

### Determination of Total Polyphenolic Content and Total Flavonoid Contents

The total polyphenolic content of the extracts prepared with solvents of different polarities, different extraction and grinding methods were determined from regression equation of calibration curve and expressed in gallic acid equivalents. In terms of solvent influence the 70% methanol extract led to the maximum TPC (32.38 ± 0.29 mg of GAE/g of dry lichen material) followed by methanol, ethyl acetate, acetone, water and hexane. These results indicate that polar solvents such as the water component of the 70% methanol and methanol assist in the extraction of polyphenolics due to the hydroxyl moieties on the phenols. With the sequential extraction the methanol extract showed the maximum TPC, followed by ethyl acetate, dichloromethane, 50% methanol and finally hexane. The low amount of TPC achieved in the 50% extract might be attributed to extraction of major polyphenol beforehand by methanol in the sequence.

Importantly, processing of the sample with different grinding methods led to separation of fractions providing one fraction rich in total polyphenol and flavonoid content. The sample ground with the mortar and pestle gave a uniform powder mixture (whole thallus) that produced a higher TPC content (32.38 ± 0.29 mg of GAE/g of dry lichen material). On the other hand the sample ground in the mixer grinder gave two separate fractions; cortex powder having almost similar TPC content (28.06 ± 0.16 mg of GAE/g of dry lichen material) as obtained in whole thallus sample and the medulla pieces providing lower TPC content (14.87 ± 0.03 mg of GAE/g of dry lichen material).

In another set of experiments, comparison of extraction of total polyphenols with the optimized extraction solvent mixture (70% Methanol) was also carried out using four commonly used extraction techniques (soxhlet, reflux, sonication and maceration). Table 1 shows the result of total polyphenol content obtained by four different extraction procedures. The results indicate that the maximum yield of total polyphenolics was achieved by soxhlet extraction followed by reflux, sonication and maceration. Exhaustive extraction of total polyphenols in soxhlet extracts is because of the repeated and continuous washing of the crude material and application of heat during extraction as compared to other extraction methods.

The total flavonoid content of the extracts prepared with solvents of different polarities, different grinding and extraction methods were determined from the regression equation of calibration curve obtained from quercetin and expressed in quercetin equivalents. The different solvents exhibited substantial differences in the TFC as depicted in Table 1. The TFC ranged from 0.02 ± 0.00 mg of QE/g of dry lichen material for hexane to 0.38 ± 0.00 mg of QE/g of dry lichen material for 70% methanol extract. In case of sequential extraction, 50% methanol extract has maximum flavonoid content (0.11 ± 0.00 mg QE/g) followed by dichloromethane, methanol and ethylacetate. The mixer grinder processing of samples led to an improved separation of fractions rich in flavonoids. The TFC content of the cortex powder fraction (0.35 ± 0.00 mg QE/g) is almost equivalent to the whole thallus sample (0.38 ± 0.00 mg QE/g) whereas medulla pieces provided a lower TFC content (0.11 ± 0.00 mg of QE/g of dry lichen material). When considering the different extraction methods the maximum TFC were afforded by soxhlet extraction (0.44 ± 0.00 mg of QE/g of dry lichen material) followed by reflux, sonication and maceration.

### Free Radical Scavenging Potential

To evaluate the free radical scavenging potential of different extracts, three types of assays were employed. The chemical composition of different extracts will have an influence depending on the test employed and therefore more than one assay was performed to get a thorough understanding of the extract’s antioxidant potential. Antioxidant potential of the different samples were expressed as µM of Trolox equivalents per gram of dry lichen material as it is a more significant and descriptive expression. The results of antioxidant potential of different extract by ABTS, DPPH and OH scavenging assay are summarized in Table 1. Among the various solvents used, extracts obtained with 70% methanol exhibit higher antioxidant potential with highest TEAC value. With the different grinding techniques, the TPC, TFC and antioxidant potential obtained from cortex powder is comparable to the homogenised powder. Separation of surface metabolites in cortex powder portions provides an enriched fraction targeted to specific metabolites and also in terms of TPC, TFC and antioxidant potential. When comparing the different extraction methods, it was also observed that the extraction of the metabolites having maximum TPC, TFC and highest antioxidant potential was found *via* soxhlet extraction.

### Antioxidant Potential

**FIGURE 3:**
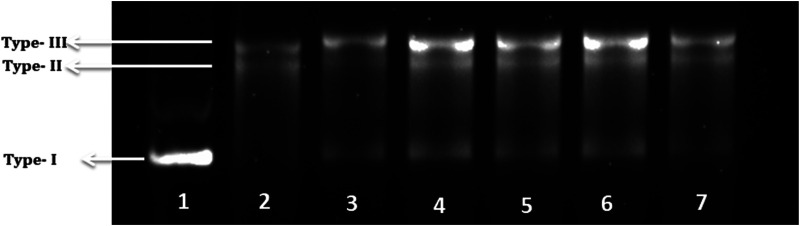
DNA Nicking assay showing protective effect of PF70M extract against hydroxyl radical generated by Fenton’s reagent. Lane 1: Native plasmid DNA pBSK without treated with Fenton’s reagent; Lane 2: DNA treated with Fenton’s reagent; Lane 3: DNA treated with Fenton’s reactant and 25 μg/ml Curcumin; Lane 4–7: DNA treated with Fenton’s reactant and *Pseudevernia furfuracea* 70% methanol extract (200, 100, 50 and 25 μg/ml, respectively).

Lastly, a DNA nicking assay was used to understand the protective effect of specific extract against hydroxyl radical’s destructive effects on DNA. Hydroxyl radicals generated using Fenton’s reaction mixture resulted in degradation of supercoiled form of plasmid DNA (Lane 1, Type 1) into single stranded nicked and double stranded linear forms of DNA (Lane 2, Type II and III) (Figure 3). Results showed that presence of curcumin in 25 μg/ml concentrations (Lane 3) protect the DNA in supercoiled form. Similarly, presence of PF70M extract at different concentrations (200–25 μg/ml, Lane 4–7, respectively) in the reaction mixture diminished the DNA damage. Thus, concentration-dependent intensification of native supercoiled DNA (Type 1) was observed.

### Correlation of Antioxidant Potential With Total Polyphenolic Content and Total Flavonoid Content

The redox potential of the components such as polyphenols, phenolic acids and flavonoids is the determining factor for the antioxidant property of any food or herbal entity (Tembo et al., 2017). A statistical significant relationship was established between total polyphenol contents, total flavonoid content and TEAC values obtained from DPPH, ABTS and hydroxyl scavenging assays (Table 2). As a correlation between the TPC and antioxidant potential are marginally better when compared to the correlation between the TFC and antioxidant potential we suggest here that the phenolic compounds play a more significant role towards antioxidant potential than the flavonoids.

**TABLE 2:** Values of Pearson’s correlation coefficients (r) for the TPC, TFC, TEAC (DPPH), TEAC (ABTS), TEAC (OH radical scavenging), Atraric acid and Olivetoric acid concentration.

Correlations	TPC content (mg GAE/DW)	TFC content (mg QE/DW)	TEAC ABTS (uM Trolox/DW)	TEAC DPPH (uM Trolox/DW)	TEAC OH radical scavenging (uM Trolox/DW)	Atraric acid conc (mg/g DW)	Olivetoric acid conc (mg/g DW)
TPC content (mg GAE/DW)	1	0.874**	0.979**	0.979**	0.962**	0.873**	0.867**
TFC content (mg QE/DW)	0.874**	1	0.842**	0.920**	0.936**	0.846**	0.701**
TEAC ABTS (µM Trolox/DW)	0.979**	0.842**	1	0.972**	0.950**	0.824**	0.870**
TEAC DPPH (µM Trolox/DW)	0.979**	0.920**	0.972**	1	0.986**	0.863**	0.819**
TEAC OH radical scavenging (µM Trolox/DW)	0.962**	0.936**	0.950**	0.986**	1	0.827**	0.808**
Atraric acid conc (mg/g^a^W)	0.873**	0.846**	0.824**	0.863**	0.827**	1	0.672**
Olivetoric acid conc (mg/g DW)	0.867**	0.701**	0.870**	0.819**	0.808**	0.672**	1

**Correlation is significant at the 0.01 level (2-tailed)

### Spatial Localization of Metabolites Using High Performance Liquid Chromatography and Scanning Electron Microscopy

#### Surface Morphology Using Scanning Electron Microscopy

**FIGURE 4:**
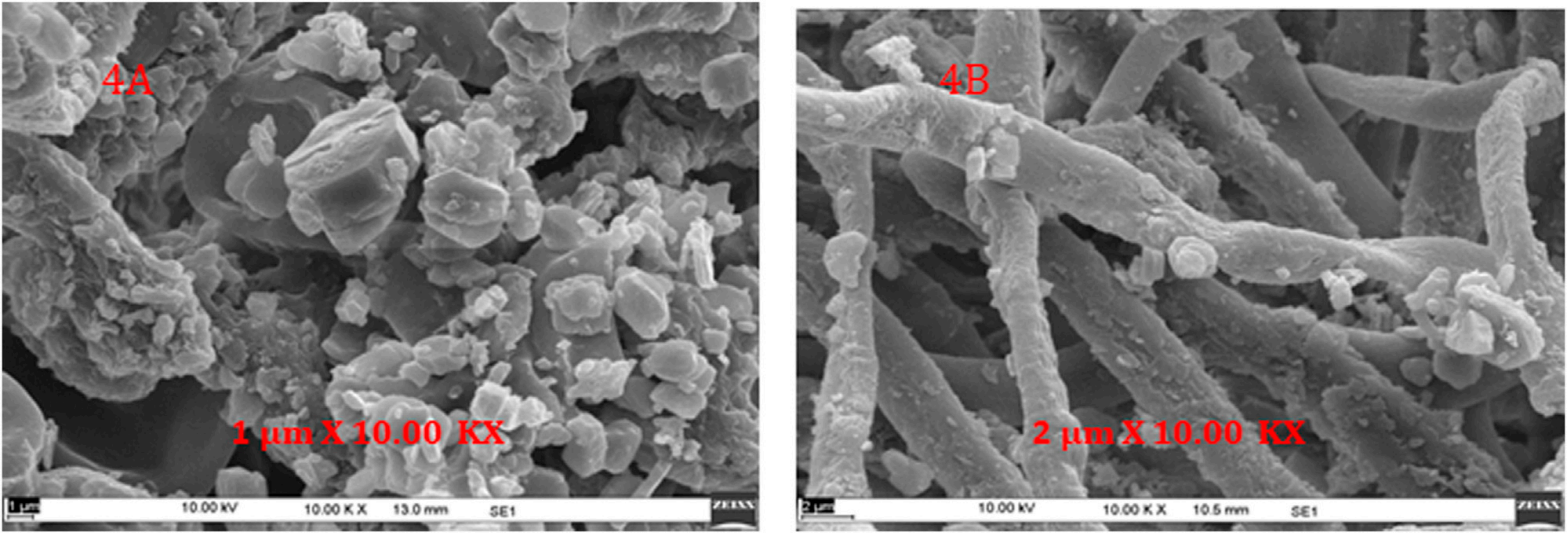
Spatial localization of metabolites using SEM, Scanning electron microscope images of sample after grinding employing mixer grinder **(A)** Cortex powder **(B)** Medulla pieces.

As discussed earlier, samples ground in the mixer grinder lead to separation of fine powder (cortex) and coarser segments (medulla). These two portions were examined using SEM (EVO MA10, Carl Zeiss) to have a clear understanding of their surface morphology. This approach also helped to understand the spatial localization of some surface metabolites in sample. Microscopic observation of the two portions identifies some crystals like structure present on the surface (Figure 4). This result is consistent with the findings of Komaty et al., who have discussed the separation of cortex powder from medulla pieces and thus obtained atranorin localized on the cortex portion of *P. furfuracea* (Komaty et al., 2016).

#### Determination of Atraric Acid and Olivetoric Acid by High Performance Liquid Chromatography

**FIGURE 5:**
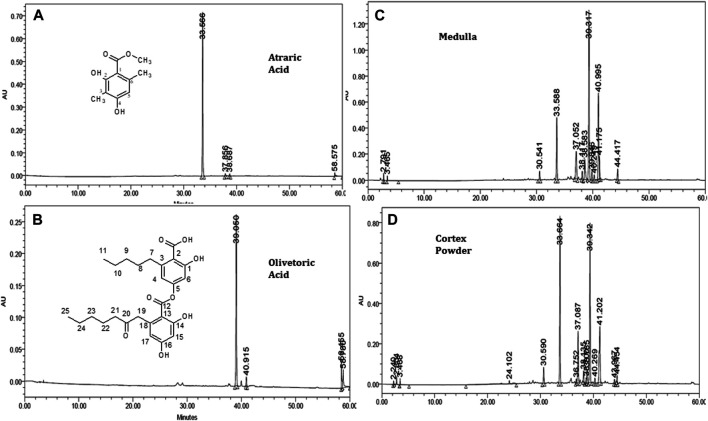
HPLC chromatogram of Atraric acid **(A)**, Olivetoric acid **(B)**, Medulla pieces **(C)**, Cortex powder **(D)**.

To further confirm this in our study, both of these portions were treated separately for extraction and identification of their respective metabolites. Characterisation of the some constituents in the extracts was performed with the two secondary metabodsllites that has been isolated namely atraric acid and olivetoric acid. These metabolites were characterized using NMR and mass spectroscopic data. Typical chromatograms of the standard compounds are shown in Figure 5. The calibration curves of atraric acid and olivetoric acid also showed good linearity (*R* ^2^ = 0.9994 and 0.9971, respectively). Quantification of these metabolites in all the extracts obtained using different extraction solvents, techniques and grinding methods was carried out using regression equation obtained from their calibration plots and results are presented in Table 1. In the case of different solvent extraction, the maximum concentration of both the compounds were found in the 70% methanol extract, matching the trend observed in total phenolic content study. With the different extraction methods, the maximum concentration of atraric acid was achieved *via* soxhlet extraction and interestingly the concentration of olivetoric acid was higher in the reflux process. This highlights the importance of taking holistic approaches towards sample extract optimisation.

The concentrations of both the compounds were observed to be higher in the homogenised powder followed by the cortex powder and the medulla. A marginal difference of olivetoric acid was found between cortex powder and medulla pieces highlighting presence of olivetoric acid in both the portions equally and cannot be separated by this method. Interstingly the concentration of the atraric acid which has been reported as hydrolysis derivative of atranorin when come in contact with methanol or processed in the presence of methanol (Oettl et al., 2014) was found to be more concentrated in the powder compared to the cortex (Figure 5) thus highlighting the surface localization of atranorin and can be easily separated from the sample by simple pre-processing.

A good correlation was found between concentration of both compounds and TEAC obtained from ABTS, DPPH and OH scavenging assay in all the three parameters (Table 2). These results indicate that these two metabolites might contribute strongly toward the antioxidant potential of this lichen.

Based on this information the importance of the lichen *Pseudevernia furfuracea* have been shown to have key metabolites that contribute strongly to the antioxidant profile of the food product. Lichens in general are underutilised in western food cuisine and this work shows that they may have an important place to in food products. In generally lichens have not been well studied in terms of their potential as a healthy food product and this highlights them as a viable avenue which could even lead to large scale production of them. For this to occur and for the successful application of them into food products it is clear that the way in which the sample is processed and if required the approach to biomolecule extraction is particularly important.

### Identification Data (Nuclear Magnetic Resonance and Mass Spectra) of Compounds

Both the compounds were identified on the basis of nuclear magnetic resonance (NMR) data and high resolution mass spectrometry (HRMS) data reported previously.

**Atraric acid (1): Pale yellow crystal,** ^1^H-NMR (CDCl_3_)δ: 2.16 (3H, s, 3-Me), 2.52 (3H, s, 6-Me), 3.95 (3H, s, -C=OOCH_3_), 6.28 (1H, s, H-5), 12.40 (1H,s, 4-OH), 12.88 (1H, s, 2-OH); ^13^C-NMR (CDCl_3_) δ: 176.54 (-C=O), 166.74 (C-4), 164.06(C-2), 143.42(C-6), 114.05(C-1), 112.45(C-3), 107.53(C-5), 54.56(C-OCH_3_), 26.86(Me-C6), 10.48(Me-C3). (+)HRMS: 197.0807 [M + H]^+^. Obtained data was in good agreement with reported literature (Gormann et al., 2003).

**Olivetoric acid (2): Pale yellow amorphous powder**, ^1^H-NMR (CDCl_3_)δ: 0.93 (6H, t, 11-Me and 25-Me), 1.3 (8H, m, H-9,10, 23, 24), 1.5 (2H, dd, H-7), 2.4 (2H, t, H-21), 2.8 (2H, bs, H-19), 6.5 (4H, d, H-4,6,15,17),10.0 (1H,s, 16-OH), 10.5 (2H,s, 1-OH, 14-OH); ^13^C-NMR (CDCl_3_) δ: 163.21 (C-14), 162.69 (C-1), 158.39 (C-16), 142.06 (C-5), 132.72 (C-18), 104.13 (C-2), 103.02 (C-13), 100.92 (C-15), (+) HRMS: 473.54 [M + H]^+^.

### Composition of 70% Methanol Extract of *Pseudevernia furfuracea* Using UHPLC-DAD-MS

**FIGURE 6:**
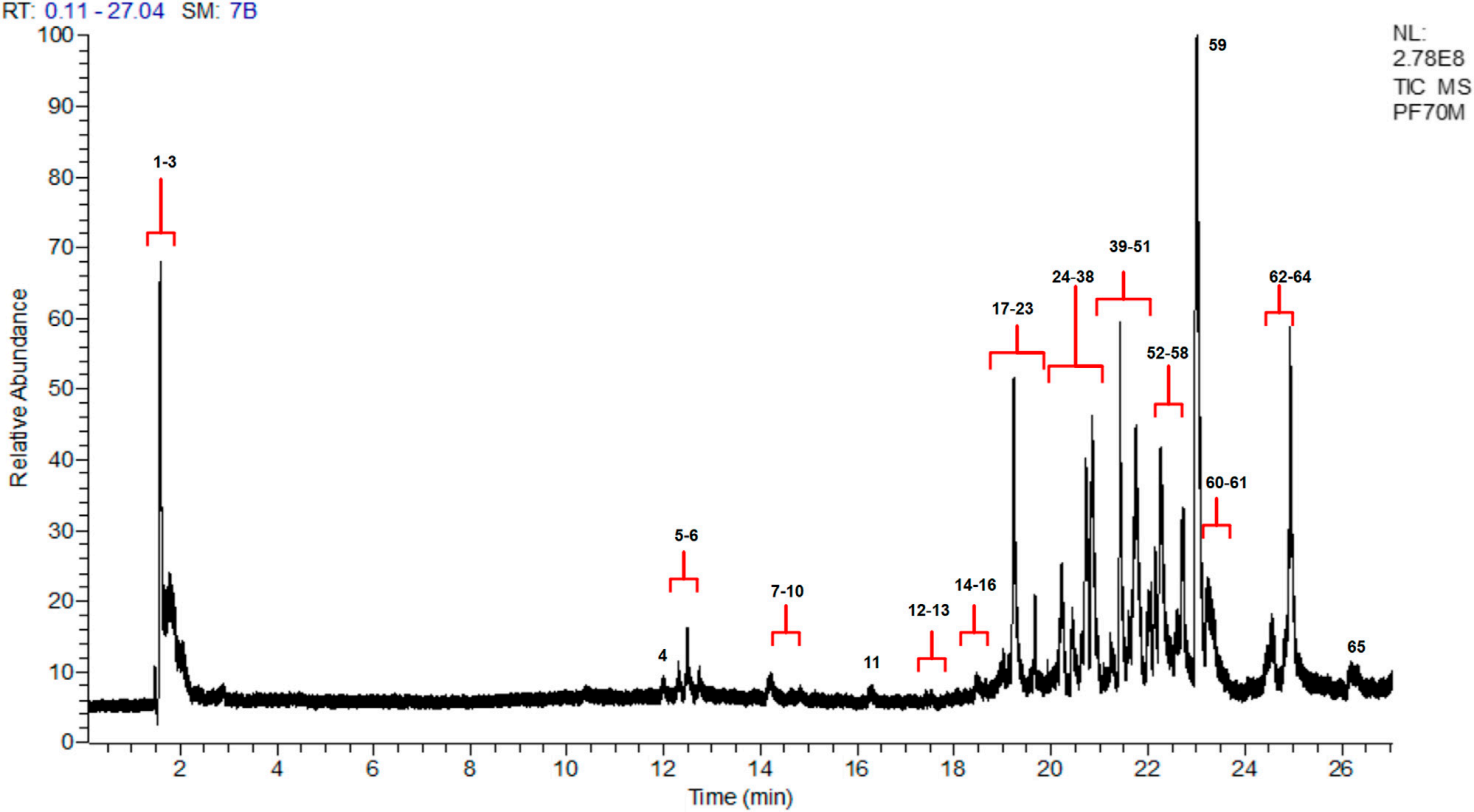
UHPLC chromatograms of *Pseudevernia furfuracea* 70% methanol extract.

Electrospray Orbitrap is a rapid and state of art tool for the characterization of metabolites in various food and herbal commodity. Considering the metabolites profiling of 70% methanol extract, it has been subjected to UHPLC/ESI/MS/MS analysis to investigate the whole biochemical composition of stated extract. Figure 6 shows the total ion current chromatograms (TIC) of 70% methanol extract and Table 3 presented the list of all the compounds tentatively identified on the basis of their *m/z* of molecular ion [M-H]^−^ and respective mass information extracted through their MS and MS/MS spectra. Sixty five peaks were detected using UHPLC/ESI/MS/MS in the negative mode. Of the forty nine compounds identified in this species the majority were depsides, depsidones, depsone, lipids, pulvinic acid derivative, diphenylether derivatives and dibenzofurans. Eight depsides moieties namely decarboxythamnolic acid, boninic acid, epiphorellic acid II, 4-O-methylolivetoric acid, dihydropicrolichenic acid, haemathamnolic acid isomer, squamatic acid and 2, 2′ -di-O-methylanziaic acid, respectively were tentatively identified. Few of these compounds are in congruent with previous reported data (Torres-Benítez et al., 2017).

**TABLE 3:** Biochemical composition of 70% methanol extract by UHPLC-DAD-MS analysis the negative ion mode.

Peak	Tentative identification	Molecular formula	Retention time (min.)	Theoretical mass (*m/z*)	Measured mass (*m/z*)	Accuracy (ppm)	Metabolite type	MS ions (ppm)
1	Arabic acid	C_5_H_9_O_6_	1.59	165.0399	165.0401	−1.2	Acid	147.0294; 113.0237; 129.0187
2	Atraric acid^a^	C_6_H_11_O_7_	1.58	195.0505	195.0508	−1.5	Acid	165.0401
3	Glucosylglycolate	C_8_H_13_O_8_	1.67	237.0610	237.0619	−3.8	Acid	207.0511; 147.0296
4	Derivative 2,4-Diformyl-3,5-dihydroxytoluene o 2,6-Diformyl-3,5-dihydroxytoluene	C_9_H_7_O_6_	11.99	211.0243	211.0247	−1.9	A	179.0349; 167.0336; 149.0239
5	Orsellinic acid	C_8_H_7_O_4_	12.31	167.0344	167.0347	−1.8	A	123.0445
6	2,4-Dicarboxy-3-hydroxy-5-methoxytoluene	C_10_H_9_O_6_	12.48	225.0399	225.0404	−2.2	A	181.0504; 167.0345; 149.0240
7	Unknown	—	14.19	—	—	—	—	371.1017 339.0755 278.1034 141.0917 135.1196
8	9,10,12,13-Tetrahydroxyhexadecanoic acid	C_16_H_31_O_6_	14.26	319.2121	319.2128	−2.2	L	301.2022
9	Methyl porphyrilate	C_17_H_11_O_7_	14.82	327.0505	327.0513	−2.4	DBF	283.0612; 181.0498
10	9,10,12,13,14-Pentahydroxytricosenoic acid	C_23_H_43_O_7_	15.18	431.3009	431.3015	−1.4	L	—
11	Hexahydroxytetracosanoic acid	C_24_H_47_O_8_	16.27	463.3271	463.3277	−1.3	L	389.1244
12	9,10,12,13-tetrahydroxynonadecanoic acid	C_19_H_37_O_6_	17.55	361.2590	361.2598	−2.2	L	—
13	Menegazziaic acid	C_18_H_13_O_9_	17.87	373.0560	373.0567	−1.9	D	329.0671; 167.0344; 149.0240; 151.0398
14	Hexahydroxytetracosenoic acid	C_24_H_45_O_8_	18.46	461.3114	461.3121	−1.5	L	417.2859; 375.5755
15	9,10,12,13,14-Pentahydroxytricosanoic acid	C_23_H_45_O_7_	18.66	433.3165	433.3171	−1.4	L	329.0671; 389.2546
16	9,10,12,13-Tetrahydroxyeicosanoic acid	C_20_H_39_O_6_	18.95	375.2747	375.2754	−1.9	L	361.2609
17	Unknown	—	19.01	—	—	—	—	467.2780 413.2912 375.2755 135.0389 119.0173
18	9,10,12,13,14-Pentahydroxytetracosanoic acid	C_24_H_47_O_7_	19.13	447.3322	447.3328	−1.3	L	433.3170
19	Unknown	—	19.22	—	—	—	—	481.2937 445.3171 389.2911 181.3367 109.1143
20	Olivetonic acid, 2,4-Dihydroxy-6-(2′-oxo-n-heptyl)-benzoic acid	C_14_H_17_O_5_	19.65	265.1076	265.1083	−2.6	A	247.0972; 221.1182
21	Loxodellonic acid	C_23_H_23_O_8_	19.74	427.1393	427.1400	−1.6	D	235.0975; 385.0932; 343.0970; 249.0767; 195.0662
22	9,10,12,13-Tetrahydroxypentacosanoic acid	C_24_H_45_O_7_	19.88	445.3165	445.3173	−1.8	L	417.3225
23	Hexahydroxyhexacosanoic acid	C_26_H_51_O_8_	19.93	491.3584	491.3589	−1.0	L	445.3173; 345.2437
24	9,10,12,13-tetrahydroxydocosanoic acid	C_22_H_43_O_6_	20.04	403.3060	403.3068	−2.0	L	375.2755
25	Unknown	—	20.13	—	—	—	—	309.0983 265.1081 237.0767 190.0540 103.0760
26	9,10,12,13-Tetrahydroxydocosanoic acid	C_22_H_43_O_6_	20.23	403.3060	403.3067	−1.7	L	387.3123
27	Unknown	—	20.25	—	—	—	—	451.2832 403.3066 345.2437 110.2839 108.2998
28	9,10,11,12,13,14,15-Hexahydroxycosaenoic acid	C_26_H_49_O_8_	20.43	489.3427	489.3432	−1.0	L	429.3227
29	Decarboxythamnolic acid	C_18_H_15_O_9_	20.55	375.0716	375.0724	−2.1	d	167.0345; 209.0455
30	9,10,12,13,14 Pentahydroxyhexacosanoic acid	C_26_H_51_O_7_	20.66	475.3635	475.3641	−1.3	L	431.3376; 447.3331
31	Hexahydroxyoxooctacosenoic acid	C_28_H_53_O_9_	20.66	533.3690	533.3694	−0.7	L	475.3640
32	9,10,12,13-tetrahidroxytricosanoic acid	C_23_H_45_O_6_	20.72	417.3216	417.3224	−1.9	L	403.3067
33	Pulvinic acid	C_18_H_11_O_5_	20.80	307.0606	307.0615	−2.9	PAD	263.0715; 117.0342
34	Boninic acid	C_25_H_31_O_8_	20.85	459.2019	459.2026	−1.5	d	415.2128; 237.1130; 281.1033; 253.1078; 223.0974
35	Unknown	—	20.85	—	—	—	—	509.3247 459.2026 415.2129 345.2437 109.2090
36	Epiphorellic acid II	C_26_H_31_O_9_	20.92	487.1968	487.1974	−1.2	d	443.2076; 235.0977; 251.0930; 429.1917
37	Loxodinol	C_25_H_29_O_9_	20.98	473.1812	473.1819	−1.5	DE	429.1920; 237.0768
38	Unknown	—	20.98	—	—	—	—	263.0927 243.0066 221.0816 209.0454 104.0931
39	Olivetolcarboxylic acid	C_12_H_15_O_4_	21.08	223.0970	223.0975	−2.2	A	179.1074; 207.1027
40	4-O-Methylolivetoric acid	C_27_H_33_O_8_	21.20	485.2175	485.2182	−1.4	d	441.2286; 279.1240
41	6-ethyl-6-n-pentylpentadecan-4,5,7,8,15-pentol-15-acetate	C_24_H_47_O_6_	21.20	431.3373	431.3381	−1.9	Aliphatic compounds	417.3233
42	Unknown	—	21.24	—	—	—	—	501.2130 485.2180 455.1588 431.3380 345.2438
43	9-Hydroxydocosapentaenoic acid	C_22_H_33_O_3_	21.32	345.2430	345.2439	−2.6	L	301.2536
44	Unknown	—	21.38	—	—	—	—	419.0988 265.1083 151.0396 123.0446 105.0337
45	Dihydropicrolichenic acid	C_25_H_31_O_7_	21.42	443.2070	443.2077	−1.6	d	399.2177; 221.1184; 151.0394
46	Haemathamnolic acid isomer	C_19_H_15_O_10_	21.51	403.0665	403.0673	−2.0	d	371.0412; 209.0455; 359.0778
47	Unknown	—	21.61	—	—	—	—	468.2028 443.2077 210.1328 144.2105 122.3307
48	Unknown	—	21.67	—	—	—	—	261.0770 233.0817 223.0975 179.1075 151.0398
49	3-Hydroxyphysodic acid	C_26_H_29_O_9_	21.69	485.1812	485.1817	−1.0	D	441.1926; 247.0976
50	Acido-nor-8′-metilconstictico	C_21_H_19_O_11_	21.69	447.0927	447.0933	−1.3	D	209.0455; 403.1052
51	Squamatic acid	C_19_H_17_O_9_	21.74	389.0873	389.0880	−1.8	d	193.0140; 163.0396; 149.0239; 119.0497; 121.0289
52	Methylphysodic acid	C_27_H_31_O_8_	22.06	483.2019	483.2023	−0.8	D	453.0593; 345.2436
53	Picrolichenic acid isomer	C_25_H_29_O_7_	22.15	441.1913	441.1920	−1.6	Depsone	—
54	Physodic acid	C_26_H_29_O_8_	22.25	469.1862	469.1866	−0.9	D	425.1969; 247.0973; 451.1762
55	Haemophaein	C_27_H_31_O_7_	22.42	467.2070	467.2076	−1.3	DBF	247.0975; 425.1970; 451.1762
56	β-Alectoronic acid	C_28_H_31_O_9_	22.60	511.1968	511.1971	−0.6	DE	369.1351; 247.0975; 263.0927; 467.2073
57	2,2′-Di-O-methylanziaic acid	C_26_H_33_O_7_	22.67	457.2226	457.2234	−1.7	d	265.1080; 413.2310; 427.2128; 443.2079
58	Physodic acid isomer	C_26_H_29_O_8_	22.73	469.1862	469.1868	−1.3	D	247.0976; 425.1968
59	Derivative physodic acid	C_25_H_29_O_6_	23.00	425.1964	425.1973	−2.1	D	381.2068; 177.0945
60	Unknown	—	23.23	—	—	—	—	423.0492 395.0543 409.0699 163.0394 119.0497
61	Vulpinic acid	C_19_H_13_O_5_	23.49	321.0763	321.0771	−2.5	PAD	—
62	Picrolichenic acid	C_25_H_29_O_7_	24.55	441.1913	441.1920	−1.6	Depsone	359.1863
63	9-Hydroxydocosapentaenoic acid isomer	C_22_H_33_O_3_	24.69	345.2430	345.2438	−2.3	L	301.2538
64	2′-O-Methylphysodone	C_26_H_31_O_6_	24.92	439.2121	439.2126	−1.1	DE	
65	Unknown	—	26.25	—	—	—	—	555.2851 537.2493 389.2700 239.2504 119.2575

^a^ Isolated, identified and quantifed.

Where A = Aromatic; L = Lipid; D = depsidone; d = depside; DE = diphenylether; DBF = dibenzofuran; PAD = pulvinic acid derivative.

Along with depsides, eight depsidones moieties were identified namely menegazziaic acid (Salgado et al., 2018), loxodellonic acid, 3-hydroxyphysodic acid, methylphysodic acid (Le Pogam et al., 2015), physodic acid, acido-nor-8′-metilconstictico, physodic acid isomer and derivative physodic acid, respectively. Two depsone moieties identified as picrolichenic acid isomer and picrolichenic acid, both having [M-H]^−^ ions at m/z 441.1920 were also detected.

Two dibenzofuran (DBF) corresponding to peak 9 and 55 were identified as methyl porphyrilate and haemophaein, respectively. Besides, DBF, two pulvinic acid derivative namely pulvinic acid and vulpininc acid corresponding peak 33 and 61 were also identified. β-Alectoronic acid and 2′-O-methylphysodone belongs to category of diphenyl ether has also been detected. Peak 37 was identified as loxodinol (diphenyl ether) (Torres-Benítez et al., 2017).

As is often the case with biological samples several components are not fully elucidated however the mass spectral information generated here forms the basis for their further investigation.

### Cytotoxicity Assay

**FIGURE 7:**
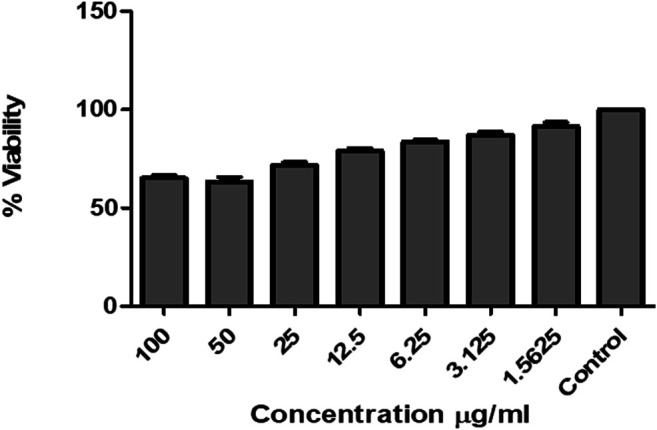
Effect of *Pseudevernia furfuracea* 70% methanol extract on HEK 293Tcell lines.

PF70M extracts were tested for their toxic effect on HEK 293T cells by *in vitro* viability test method using Alamar blue as described in methods. The data obtained was plotted as bar graph depicting the percent difference between treated and un-treated cells to evaluate the level of cellular viability (Figure 7). The results showed that after treatment with varying concentrations (0.156 –100 μg/ml) of PF70M extracts there was no significant cell death as compared to control cells as shown in Figure 7 suggesting its safe application to host cells.

### Conclusion

The chemical composition of lichen extracts mainly depends upon the method used for the extraction of metabolites. Studying various extraction processes using different solvents, methods and pre-processing techniques, we have found that 70% methanol extract using soxhlet method and mixer grinder technique is the most efficient combination for the enrichment of powder fraction targeting specific metabolites. Extract obtained using this combination resulted in a fraction rich in polyphenolic and flavonoids compounds that accounted for the significant antioxidant potential. The findings of the present work suggest that this lichen used in the form of spice mixture has also potential antioxidant effect. Also the safety of the active extract could be proved with the cytotoxicity and DNA damage protection assays. The antioxidant potential of the detected metabolites obtained from profiling of the active extract should be further explored in suitable cell based and *in vivo* models. Also, detailed studies for elucidation of mechanism behind these activities can further contribute in the better utilization of this lichen.

## Data Availability

The raw data supporting the conclusions of this article will be made available by the authors, without undue reservation, to any qualified researcher.

## References

[B1] Aoussar N. Manzali R. Nattah I. Rhallabi N. Vasiljevic P. Bouksaim M. (2017). Chemical composition and antioxidant activity of two lichens species *(Pseudevernia furfuracea* L and *Evernia prunastri* L) collected from Morocco. J. Mater. Environ. Sci. 8 (6), 1968–1976.

[B2] Brand-Williams W. Cuvelier M. E. Berset C. (1995). Use of a free radical method to evaluate antioxidant activity. LWT - Food Sci. Technology 28 (1), 25–30. 10.1016/S0023-6438(95)80008-5

[B13] Cacace J. E. Mazza G. (2003). Optimization of extraction of anthocyanins from black currants with aqueous ethanol. J food sci. 68 (1), 240–248. 10.1111/j.1365-2621.2003.tb14146.x

[B3] Calcott M. J. Ackerley D. F. Knight A. Keyzers R. A. Owen J. G. (2018). Secondary metabolism in the lichen symbiosis. Chem. Soc. Rev. 47 (5), 1730–1760. 10.1039/c7cs00431a 29094129

[B4] Chowdhary K. Kaushik N. (2019). UPLC-MS and dereplication-based identification of metabolites in antifungal extracts of fungal endophytes. Proc. Natl. Acad. Sci. India, Sect. B Biol. Sci. 89 (4), 1379–1387. 10.1007/s40011-018-1060-3

[B5] Crittenden P. Porter N. (1991). Lichen-forming fungi: potential sources of novel metabolites. Trends Biotechnol. 9 (1), 409–414. 10.1016/0167-7799(91)90141-4 1367745

[B6] Goel M. Kalra R. Ponnan P. Jayaweera J. A. A. S. Kumbukgolla W. W. (2021). Inhibition of penicillin-binding protein 2a (PBP2a) in methicillin resistant Staphylococcus aureus (MRSA) by combination of oxacillin and a bioactive compound from Ramalinaroesleri. Microb. Pathogenesis 150, 104676. 10.1016/j.micpath.2020.104676 33278518

[B7] Goel M. Rani A. Dureja P. Uniyal P. (2014). Investigation of allelopathic potentiality of the himalyan lichen *parmelia reticulata* tayl. Against *phalaris minor* retz. APCBEE Proced. 9, 140–144. 10.1016/j.apcbee.2014.01.025

[B8] Goel M. Sharma P. K. Dureja P. Rani A. Uniyal P. L. (2011). Antifungal activity of extracts of the lichensParmelia reticulata, Ramalina roesleri, Usnea longissimaandStereocaulon himalayense. Arch. Phytopathology Plant Prot. 44 (13), 1300–1311. 10.1080/03235408.2010.496549

[B9] Gormann R. Kaloga M. Li X.-C. Ferreira D. Bergenthal D. Kolodziej H. (2003). Furanonaphthoquinones, atraric acid and a benzofuran from the stem barks of *Newbouldia laevis* . Phytochemistry 64 (2), 583–587. 10.1016/s0031-9422(03)00277-2 12943779

[B10] Güvenç A. Küpeli Akkol E. Süntar İ. Keleş H. Yıldız S. Çalış İ. (2012). Biological activities of *Pseudevernia furfuracea* (L.) Zopf extracts and isolation of the active compounds. J. Ethnopharmacology 144 (3), 726–734. 10.1016/j.jep.2012.10.021 23107822

[B11] Hidalgo M. E. Ferna´ndez E. Quilhot W. Lissi E. (1994). Antioxidant activity of depsides and depsidones. Phytochemistry 37 (6), 1585–1587. 10.1016/s0031-9422(00)89571-0 7765999

[B12] Ismail B. B. Pu Y. Guo M. Ma X. Liu D. (2019). LC-MS/QTOF identification of phytochemicals and the effects of solvents on phenolic constituents and antioxidant activity of baobab (*Adansonia digitata*) fruit pulp. Food Chem. 277, 279–288. 10.1016/j.foodchem.2018.10.056 30502146

[B14] Komaty S. Letertre M. Dang H. D. Jungnickel H. Laux P. Luch A. (2016). Sample preparation for an optimized extraction of localized metabolites in lichens: application to *Pseudevernia furfuracea* . Talanta 150, 525–530. 10.1016/j.talanta.2015.12.081 26838439

[B15] Kosanic M. Manojlovic N. Jankovic S. Stanojkovic T. Rankovic B. (2013). *Evernia prunastri* and *Pseudoevernia furfuraceae* lichens and their major metabolites as antioxidant, antimicrobial and anticancer agents. Food Chem. Toxicol. 53, 112–118. 10.1016/j.fct.2012.11.034 23220145

[B16] Kosanić M. Ranković B. Stanojković T. Rančić A. Manojlović N. (2014). Cladonia lichens and their major metabolites as possible natural antioxidant, antimicrobial and anticancer agents. LWT Food Sci.Technol. 59 (1), 518–525. 10.1016/j.lwt.2014.04.047

[B17] Kumar J. Dhar P. Tayade A. B. Gupta D. Chaurasia O. P. Upreti D. K. (2014). Antioxidant capacities, phenolic profile and cytotoxic effects of saxicolous lichens from trans-Himalayan cold desert of Ladakh. PLoS One 9 (6), e98696. 10.1371/journal.pone.0098696 24937759 PMC4061001

[B18] Le Pogam P. Schinkovitz A. Legouin B. Le Lamer A.-C. Boustie J. Richomme P. (2015). Matrix-free UV-laser desorption ionization mass spectrometry as a versatile approach for accelerating dereplication studies on lichens. Anal. Chem. 87 (20), 10421–10428. 10.1021/acs.analchem.5b02531 26378462

[B19] Li X. (2013). Solvent effects and improvements in the deoxyribose degradation assay for hydroxyl radical-scavenging. Food Chem. 141 (3), 2083–2088. 10.1016/j.foodchem.2013.05.084 23870931

[B20] Liu R. H. (2003). Health benefits of fruit and vegetables are from additive and synergistic combinations of phytochemicals. Am. J. Clin. Nutr. 78 (3 Suppl. l), 517S–520S. 10.1093/ajcn/78.3.517S 12936943

[B21] Manojlovic N. Rankovic B. Kosanic M. Vasiljevic P. Stanojkovic T. (2012). Chemical composition of three Parmelia lichens and antioxidant, antimicrobial and cytotoxic activities of some their major metabolites. Phytomed 19 (13), 1166–1172. 10.1016/j.phymed.2012.07.012 22921748

[B22] Nash T. H. (1996). Lichen biology. Cambridge, U. K: Cambridge University Press.

[B23] Newman D. J. Cragg G. M. (2016). Natural products as sources of New drugs from 1981 to 2014. J. Nat. Prod. 79 (3), 629–661. 10.1021/acs.jnatprod.5b01055 26852623

[B24] Ng L. Ang Y. Khoo H. Yim H. (2012). Influence of different extraction parameters on antioxidant properties of *Carica papaya* peel and seed. Res. J. Phytochem. 6 (3), 61–74. 10.3923/rjphyto.2012.61.74

[B25] Odabasoglu F. Cakir A. Suleyman H. Aslan A. Bayir Y. Halici M. (2006). Gastroprotective and antioxidant effects of usnic acid on indomethacin-induced gastric ulcer in rats. J. Ethnopharmacology 103 (1), 59–65. 10.1016/j.jep.2005.06.043 16169175

[B38] Oettl S. K. Hubert J. Nuzillard J. M. Stuppner H. Renault J. H. Rollinger J. M. (2014). Dereplication of depsides from the lichen *Pseudevernia furfuracea* by centrifugal partition chromatography combined to 13C nuclear magnetic resonance pattern recognition. Anal. Chim. Acta 846, 60–67. 10.1016/j.aca.2014.07.009 25220142

[B26] Ordonez A. Gomez J. Vattuone M. Lsla M. (2006). Antioxidant activities of *Sechium edule* (jacq.) swartz extracts. Food Chem. 97 (3), 452–458. 10.1016/j.foodchem.2005.05.024

[B27] Rampersad S. N. (2012). Multiple applications of Alamar Blue as an indicator of metabolic function and cellular health in cell viability bioassays. Sensors 12 (9), 12347–12360. 10.3390/s120912347 23112716 PMC3478843

[B28] Re R. Pellegrini N. Proteggente A. Pannala A. Yang M. Rice-Evans C. (1999). Antioxidant activity applying an improved ABTS radical cation decolorization assay. Free Radic. Biol. Med. 26 (9-10), 1231–1237. 10.1016/S0891-5849(98)00315-3 10381194

[B29] Salgado F. Albornoz L. Cortéz C. Stashenko E. Urrea-Vallejo K. Nagles E. (2018). Secondary metabolite profiling of species of the genus *Usnea* by UHPLC-ESI-OT-MS-MS. Molecules 23 (1), 54. 10.3390/molecules23010054 PMC6017147 29280946

[B30] Singleton V. L. Orthofer R. Lamuela-Raventós R. M. (1999). [14] Analysis of total phenols and other oxidation substrates and antioxidants by means of folin-ciocalteu reagent. Methods Enzymol. 299, 152–178. 10.1016/S0076-6879(99)99017-1

[B31] Sisodia R. Geol M. Verma S. Rani A. Dureja P. (2013). Antibacterial and antioxidant activity of lichen speciesRamalina roesleri. Nat. Product. Res. 27 (23), 2235–2239. 10.1080/14786419.2013.811410 23822758

[B32] Spigno G. De Faveri D. M. (2007). Antioxidants from grape stalks and marc: influence of extraction procedure on yield, purity and antioxidant power of the extracts. J. Food Eng. 78 (3), 793–801. 10.1016/j.jfoodeng.2005.11.020

[B33] Tembo D. T. Holmes M. J. Marshall L. J. (2017). Effect of thermal treatment and storage on bioactive compounds, organic acids and antioxidant activity of baobab fruit (*Adansonia digitata*) pulp from Malawi. J. Food Compost. Anal. 58, 40–51. 10.1016/j.jfca.2017.01.002

[B34] Torres-Benítez A. Rivera-Montalvo M. Sepúlveda B. Castro O. Nagles E. Simirgiotis M. (2017). Metabolomic analysis of two *parmotrema* lichens: *P. robustum* (degel.) hale and *P. andinum* (mull. Arg.) hale using UHPLC-ESI-OT-MS-MS. Molecules 22 (11), 1861. 10.3390/molecules22111861 PMC6150355 29084151

[B35] Upreti D. K. Divakar P. K. Nayaka S. (2005). Commercial and Ethnic Use of Lichens in India. Econ. Bot. 59 (3), 269-273. 10.1663/0013-0001(2005)059[0269:caeuol]2.0.co;2

[B36] Zhao J. Ma D. Luo M. Wang W. Zhao C. Zu Y. (2014). *In vitro* antioxidant activities and antioxidant enzyme activities in HepG2 cells and main active compounds of endophytic fungus from pigeon pea [*Cajanus cajan* (L.) Millsp.]. Food Res. Int. 56, 243–251. 10.1016/j.foodres.2013.12.028

[B37] Zugic A. Jeremic I. Isakovic A. Arsic I. Savic S. Tadic V. (2016). Evaluation of anticancer and antioxidant activity of a commercially available CO2 supercritical extract of old man's beard (usnea barbata). PLoS One 11, e0146342. 10.1371/10.1371/journal.pone.0146342 26745885 PMC4706385

